# Progress in Dermatology

**Published:** 1894-05-19

**Authors:** 


### Medical Progress.

### PROGRESS IN DERMATOLOGY.

*Thyroid Feeding in Skin Diseases.*—Although Dr. Byrom Bramwell[1] has reported excellent results from thyroid feeding in psoriasis, this method does not appear to have been so satisfactory in the hands of other dermatologists. Dr. Bramwell treated three cases of psoriasis with five-drop daily doses of Brady and Martin's extract, with the result that two were quickly cured, while the third got rather worse. Dr. Talfourd Jones,[2] however, records a case of psoriasis which he treated with five-grain tabloids of thyroid extract given four times a day, in which no improvement occurred in the skin affection, but the treatment was followed by loss of flesh and cardiac failure. Dr. Squire[3] also treated two cases with thyroid extract (large doses) without any improvement. Dr. Abraham,[4] after reviewing other published cases, gives an account of sixty-five cases of psoriasis treated with thyroid feeding. Out of these improvement was noticed in 18 cases only, while disagreeable symptoms, such as headache, palpitation, muscular tremor, &c., were complained of in 28 cases. Besides the cases of psoriasis, 5 cases of lichen planus, and 7 cases of chronic eczema were treated, with benefit in about one-half of the cases. Dr. Abraham recommends three five-grain tabloids daily. A case of syphilitic psoriasis cured by thyroid feeding is reported by Dr. Gordon.[5]

*Eczema.*—Dr. Wende[6] regards infantile eczema as a purely local disease, due to want of care and attention to the child's skin. The use of ordinary cold cream is condemned, and a mixture of lanoline and vaseline (3 to 1) substituted. Since bad soap is one of the most common causes of eczema, particular care should be taken in selecting this article. Among the best soaps are lanoline and Unna's superfatted soaps. For underwear silk is the best, but finest linen or cotton may be used; woollen garments should be worn over this, but not next to the skin. Attention must also be paid to diet. Locally the eczema should be treated, after removing the crust, with carbolized liquid vaseline, by the application of calamine lotion, or of the following ointment (℞. Emplastri plumbi-simpl: vaseline a.a. m: fiat. unguent.).

Dr. Duhring[7] praises the use of tar for eczema, but its use requires great skill and judgment. In twenty-four hours it will be apparent whether tar is likely to be useful; it is certainly contra-indicated in the acute œdematous forms of eczema. The official tar ointment is too strong, and should be freely diluted; to be efficient it should be rubbed in for about five minutes; in some cases, however, a tar lotion is borne better than the ointment.

With regard to internal remedies for vesicular eczema Dr. Leslie Phillips[8] has tried calcium sulphide and calcium chloride with disappointing results. Ichthyol pills occasionally were of benefit, but the use of tartarated antimony in doses of one-tenth to one-sixth of a grain thrice daily seemed helpful in no small proportion of cases, and its continued use did not give rise to any unpleasant symptoms. Thyroid feeding gave negative results.

*Seborrhœic Eczema.*—After a histological investigation, Dr. Elliott[9] classes lichen annulatus with seborrhœic eczema, and confirms the opinion of Unna that the disease is of an inflammatory nature, and not simply a disorder of secretion. The scabs are epidermic in origin, and not derived from sebaceous gland cells, and the inflammation is not due to fat decomposition, but is probably parasitic. Dr. Jamieson[10] and Mr. Marret Tims[11] in a summary of Unna's latest paper on seborrhœic eczema, state that all forms are in reality inflammatory catarrhs of the skin. The disease is characterised by four features: (1) Parakeratosis, *i.e.*, the change which produces desquamation; (2) a tendency to mitotic proliferation of the epithelium; (3) inflammation of the cutis; (4) indications of increased activity of the coil glands with increase of the cutaneous fat. Professor Unna states that the coil of the sweat glands is permeated with fat granules, the epithelium is large with mitosis of the gland cells, and there is dilation of the lumen of the gland. A peculiar micro-organism, the morococcus (so-called from its tendency to mulberry-like aggregation) is found in the affected parts, and is accredited with producing this disorder; the coccus requires oxygen for its growth, and does not flourish deep in the skin, but is found mostly under the crusts; it has also been found in other parakeratoses and eczemas. Clinically eczema seborrhoicum spreads from the crown downwards, and is very common over the sternal, the infra clavicular, and the inter scapular regions. It tends to peripheral advance and central cure especially on the trunk; perspiration, and the use of the woollen underclothing not only favours auto-inoculation, but aggravates the symptoms. For treatment, in simple pityriasis of the scalp, a sulphur pomade (strength 1 to 9 p.c.) should be rubbed in twice a week after the scabs have been removed by spiritus saponis kalini. Later the following paste is rubbed in twice daily ℞. Zinci. oxide. 6 parts, sulphuris præcipitati 4 parts, kaolin 2 parts, adipi benzoati 28 parts. If there is much epithelial proliferation the following should be used till the reaction of the skin to chysarobin is distinct. ℞. Chysarobini 5 parts, acidi salicylici 2 parts, icthyol 5 parts, vaseline 88 parts, m. fiat unguentum.

When the itching is extreme this lotion diluted with four parts of water is soothing: ℞. Resorcini, glycerini a.a. 10 parts, spiritus 180 parts, m.

Seborrhœic eczema may sometimes simulate syphilis or psoriasis, as in a case reported by Dr. Stewart Stirling.[12]

*Prickly Heat.*—Dr. Pollitzer[13] describes this disease as a form of miliaria, and negatives its relation to eczema both on pathological and clinical grounds. Pathologically the horny layer is swollen, the rete malphigii œdematous with cystic dilatation of sweat ducts, and the cutis slightly congested. It follows that blockage of the sweat ducts is the cause of the disease. The fact that cells filled with fat do not swell up when impregnated with sweat explains the freedom of the negro from the complaint on account of his oily skin; with an Englishman on the contrary the frequent use of soap and water with frequent baths reduces the epidermic fat to a minimum, and so renders him very liable to prickly heat. As a prophylactic the body should be anointed with lanoline and almond oil after the bath, but when the rash is developed dusting powders answer best. Dr. Jamieson[14] also comments on this subject.

*Pruritus.*—Dr. Bronson,[15] in describing the treatment of this complaint, considers the condition one chiefly of hyperæsthesia. He divides treatment into (*a*) measures to remove local excitants; (*b*) sedatives, of these the internal remedies are disappointing—bromides, cannabis indica, and gelsemium are sometimes useful, but narcotics should not be used; locally carbolic acid is the most useful; (*c*) sensory stimulants (galvanism or faradism being the best); (*d*) cutaneous alteratives, including not only absorbent remedies to remove the products and control the processes of incidental inflammation, but also agents which control the blood supply, such as jaborandi; (*e*) motor depressants, such as atropin, which acts by producing muscular relaxation and vascular dilatation. Dr. Iwanow,[16] believing that prurigo is due to a want of fat in the diet, gives either lard or cod liver oil in doses of from two to four tablespoonfuls daily. Locally he uses the following lotion after a warm bath. ℞ Acid carbolic 5 parts, acid acetic 1 part, aqua or glycerine 10 parts. m. The treatment must be kept up for years to prevent relapses.

*Mould Fungi Parasitic in Man.*—Dr. Leslie Roberts[17] describes a French, English, and Austrian variety of trichophyton, and includes in the group the microsporon furfurans. In cultivations of these organisms the presence of phosphate of soda is advantageous. The different degrees of inflammation of the scalp in children is due to the variety of tricophyton present, and these affect different people in an unequal manner; thus an Austrian child is unaffected by the Austrian trichophyton, which produces a severe form of ringworm in an English child. With regard to treatment, Moir[18] recommends the application of hot antiseptic solutions to the affected area. The hair having been cut quite close, and the patch thoroughly scrubbed with soap, a pad of gauze soaked in a 1 in 2,000 solution of mercuric chloride (at 50 deg. C.) is applied; over this is placed oiled silk and a bandage. The dressing is changed daily, and the majority of the cases are cured without resort to epilation. Dr. C. Fox[19] considers that chief difficulty in the treatment lies in the situation of the fungus, and the structure of the follicle, the fungus filling the space between the hair and its internal root sheath. Treatment should be mechanical, parasiticidal, and irritative. Epilation should be done—(1) Before the hairs have degenerated too much; (2) when they have recovered their ordinary consistence; and (3) when they can be loosened by slight traction. The head should be shaved, and the form of parasiticide must be selected after careful trials; in obstinate patches croton oil is recommended. Dr. Bodin[20] cures pityriasis versicolor within eight to ten days in the following way. In the morning the parts are washed with soap and water, and in the evening the following ointment is applied: ℞. resorcini, acid salicylici, a.a. one part, sulphuris præcipitati five parts, lanoline, vaseline, and sebi. a.a. 25 parts m. Steam disinfection of the clothes is desirable to prevent recurrence.

Sycosis, according to Dr. Dumesnil,[21] must be divided into two classes, the coccogenic and the bacillogenic, the former being due to the staphylococcus aureus or albus, the latter to the bacillus sycosiferus fœtidus. The disease is contracted by dirty razors as well as by unclean towels, and may originate even by the absorption into an abraded hair follicle of micrococci floating in the air. The treatment consists in immediate and repeated epilation, combined with the use of a 1 in 500 mercuric chloride lotion every morning, and an application of camphor phénique every evening till cured. To prevent recurrence, a mercuric chloride lotion should be used after shaving, and lanoline rubbed in at night.

*Alopecia.*—Hallopeau[22] recommends essence of wintergreen for this complaint. Equal parts of the essence and ether should be rubbed in daily. Inflammation of the integument is produced with arrest of the growth of the parasites. The application is painless, and the patches so treated healed quickly. Ferraton[23] recommends that after shaving the head the patches should be covered with iodised collodion, the application being repeated after four or five days. When the collodion is stripped off after three or four applications hairs are brought away with it. A case is reported as completely cured within three months.

*Trichiasis, &c.*—Morrison[24] recommends the removal of superfluous hairs by a depilatory instead of by electrolysis, which, however, is still recommended for those strong hairs growing from moles. Equal parts of quicklime and sulphate of arsenic are made into a paste with hot water; this is allowed to dry on the affected part.

Dr. L. Guthrie[25] describes the condition of ingrowing hairs, which usually arises in consequence of shaving, in the form of bluish pimples. The cause appears to be that in shaving a hair is broken off so short that the mouth of the hair follicle closes before the hair grows sufficiently to reach it. The hair being unable to get out of the follicle coils upon itself. For treatment, the epidermis is incised, and the hair removed with forceps.

*Treatment of Lupus.*—Brocq[25] believes that lupus eythematosus can be cured by the following internal treatment: ℞ Phosphorated oil one part, cod liver oil nine parts; m. one to four teaspoonfuls a day. Moreau[27] discusses the treatment by hypodermic injection of the following substances: ℞ Thymol 3 grains, guaiacoland sterilised oil, a.a. ʒii. m.; 15 to 45 minim. are injected twice weekly into the skin of the arm or back. The injection is followed by general and local reaction. The former consists of a burning pain radiating all over the body, with a minute rise of temperature, followed by headache and transient paresis of the extremity injected, while the lupus presents signs of irritation and exudation. After six or seven injections the lupus begins to cicatrise, and the nodules pale and become depressed. This treatment is supplemented by cauterisation of the patch with a fine thermo cautery knife. It is stated that in some patients the injection gives rise to acute pulmonary congestion.

*Acne.*—Jamieson[28] recommends the following treatment for acne. For mild cases of acne rosacea, a lotion (composed of potassi sulphurat ʒi., zinci sulphati ʒi., glycerine ʒi., sp. vini. rect. ʒii., aqua ℥iv.) is applied to the face at night and washed off in the morning. This is followed by a dusting powder, composed of: ℞ acidi borici, zinci oxidi, a.a. 10 parts; talc 80 parts, bit. armen., q.s.; m. *fiat pulv. colorata*, In the case of acne indurata, Unna's salicylic acid and creosote plasters are useful, and a strong resorcin paste may also be tried. General treatment must also be employed. Philippson[29] recommends the early opening and plugging of all minute abscesses. He divides the remedies into three classes: (1) Fat dissolving substances, such as ether, chloroform, or bisulphide of carbon; (2) cosmetics; (3) acids. After the use of the first group recurrence is likely to take place.

*New Remedies.*—Gallanol,[30] which is the anilide of gallic acid, is recommended for the treatment of psoriasis and eczema. It is used either in the form of an ointment (strength, gr. xv. to ʒii. s.s. in ℥i.), or as a powder mixed with talc. The substance is non-toxic and non-irritating, but has antiseptic properties. It is best suited for mild cases of psoriasis, especially those on the face or scalp, but in severe cases it acts less rapidly than chysarobin. Twenty cases of eczema, which were treated with gallanol were cured in a short time.

Thiol[31] in the form of an ointment (four parts of thiol to thirty parts of lanoline) is useful for eczema and acne; it may also be given internally in ½ to 15 grain doses. It gives rise to no disagreeable aftertaste, nor does it disturb digestion.

The internal administration of menthol[32] in 25-grain doses is recommended for those cases of acne accompanied by indican in the urine. When the intestinal decomposition is stopped by the menthol, the indican disappears from the urine, and the acne also subsides.

Dermatol[33] has been credited by Dr. Mathews with producing a form of acute dermatitis. He had seen three cases, one, in which the powder was applied to an ulcer of leg, exhibited considerable general disturbance.

*Miscellaneous.*—In a recent paper, Professor Unna[34] calls attention to the development of carcinoma from nævi, and states that most pigmented tumours of the skin are alveolar carcinomata. From examination of nævi in the fœtus, he finds that the special "nævus cells" are derived from the rete mucosum or from the epithelium of the sebaceous glands; it is these cells which under irritation may produce a malignant tumour.

Dr. Sangster[35] and Mr. Malcom Morris[36] have lately contributed general articles on skin diseases; the former a clinical lecture on diagnosis, and the latter an address on the dermatology of to-day.

[1] Brit. Med. Journ., Oct, 28, 1893, p. 933. [2] Ibid., Dec. 30, 1893, p. 1424. [3] Ibid., Jan. 6, 1894, p. 13. [4] Lancet, Jan. 13, 1894, p. 94. [5] Brit. Med. Journ., Jan. 27, 1894. [6] Int. Clinics, vol. iii., 1893, p. 185. [7] Ibid., vol. iv., 1893, p. 352. [8] Brit. Med. Journ., Jan. 27, 1894. [9] Brit. Journ. Dermat., Dec., 1893, p. 381. [10] Ed. Med. Journ., Jan., 1894. [11] Brit. Journ. Dermat., Jan., 1894. [12] Brit. Med. Journ., Jan. 13, 1894, p. 60. [13] New York Med. Journ., Jan. 6, 1894. [14] Ed. Med. Journ., Dec., 1893. p. 563. [15] New York Med. Review, Oct, 14, 1893; Ed. Med. Journ., Jan., 1894., p. 661. [16] Wiestuck Hygieny t. Mediciny, vol. xv., No. 1. [17] Brit. Med. Journ., Jan. 27, 1894, p. 193. [18] Lancet, Dec. 23, 1893, p. 1,601. [19] Brit. Journ. Dermat., Nov., 1893, p. 268, [20] Brit. Med. Journ., Dec. 30, 1893, p. 108. [21] Int. Clinics, vol. iii., p. 357. [22] Ed. Med. Journ., Nov. 2, 1893. [23] Brit. Med. Journ., Ep., Jan. 6, 1894, p. 3. [24] Ibid. Ibid. [25] Practitioner, Jan., 1894, p. 20. [26] Med. Week, Nov. 29, 1893. [27] Ibid., Dec. 15, 1893, p. 603. [28] Brit. Journ. Dermat., Jan., 1894. [29] Fortschaff der. Med., Jan. 15, 1894. [30] Ann. de Dermatolog. et. Syph., Nov., 1893; Journ. des Maladies Cut. et Syph., July, 1893. [31] New York Med. Journ., Dec. 23, 1893, p. 776. [32] Lancet, Jan., 1894, p. 66. [33] Amer. Journ. Med. Scien., Jan., 1894, p. 93. [34] Berlin Klin. Wock., Nov., 1893; Med. Chron., Jan., 1894, p. 270. [35] Practitioner, Jan., 1894, p. 2. [36] Brit. Med. Journ., Jan. 27, 1894, p. 179.

